# Academic Burden and Emotional Problems Among Adolescents: A Longitudinal Mediation Analysis

**DOI:** 10.1002/jad.12471

**Published:** 2025-01-21

**Authors:** Jingyi Wang, Ziyao Wang, Yuting Yang, Tingting Wang, Haijiang Lin, Wei Zhang, Xiaoxiao Chen, Chaowei Fu

**Affiliations:** ^1^ School of Public Health, NHC Key Laboratory of Health Technology Assessment Fudan University Shanghai China; ^2^ Taizhou City Center for Disease Control and Prevention Taizhou China

**Keywords:** academic burden, adolescence, emotional problems, loneliness, physical activity, sleep

## Abstract

**Introduction:**

Existing research indicates high prevalence of emotional problems among adolescents with excessive academic burden, yet the underlying reasons are not well understood. This study aimed to explore loneliness, physical activity, and sleep as potential mediating pathways between academic burden and emotional problems in adolescents.

**Methods:**

A longitudinal cohort study was conducted among middle and high school students in Taizhou City, Zhejiang Province, China, with data collected at three time points. The study included 2965 adolescents, with a mean age of 15.2 years (SD = 1.7), of whom 48.0% were female. Most participants came from families with middle to high economic status (94.8%). Structural equation modeling was employed to analyze the direct associations between academic burden (measured by study time and academic stress) and depressive and anxiety symptoms. Additionally, the indirect associations were explored through three mediators: loneliness, physical activity, and sleep.

**Results:**

Higher academic stress at T1 was directly associated with more severe depressive symptoms at T3. Sleep (indirect effect 0.11, 95% CI 0.09–0.13), loneliness (0.10, 0.08–0.11) and physical activity (0.01, 0.002–0.012) at T2 mediated the relationship, accounting for 31.0%, 26.8%, and 1.8% of the total association of academic stress, respectively. For anxiety symptoms, sleep (0.11, 0.09–0.14) and loneliness (0.07, 0.05–0.08) mediated the association of academic stress with longitudinal mediation effect sizes of 34.1% and 20.6%, respectively. Study time was only associated with the outcomes indirectly via academic stress.

**Conclusions:**

Our results highlight the importance of behavioral and psychosocial differences related to academic burden in understanding the severity of mental health problems in adolescents.

## 1 Introduction

Globally, approximately one in seven adolescents aged 10–19 are affected by mental disorders, with depression and anxiety disorders collectively accounting for around 40% of cases (UNICEF DATA 2021). In China, mental health issues among adolescents are widespread, with the prevalence of depressive and anxiety symptoms reported at 24.3% and 31.6% respectively (UNICEF China 2021; Wang, Zhang, and Zhang 2020; Wang et al. 2023). These unresolved mental health challenges can significantly diminish quality of life and may persist into adulthood, exerting profound and lasting effects (Kieling et al. 2011).

One influential model explaining deteriorating adolescent mental health is the “educational stressors hypothesis,” which emphasizes the role of school‐related stress in increasing psychological distress among adolescents (West and Sweeting 2003). This hypothesis posits that adolescents' future social standing is increasingly tied to their educational performance due to shifts toward knowledge economies and expanded higher education opportunities (Högberg 2021; West and Sweeting 2003). Consequently, various school‐related stressors emerge, contributing to mental health adversities (Aanesen, Meland, and Torp 2017; Högberg 2021; Högberg, Strandh, and Hagquist 2020). Over the past four decades, China has witnessed rapid socio‐economic development, transition from industrial to knowledge economies, substantial educational expansion, and a booming off‐campus tutoring industry. All these changes have created a highly competitive educational environment. There are growing concerns about the impact of the heavy educational burden on mental health in Chinese adolescents. Studies have reported that the majority of students in China felt high or too much academic pressure, worried a lot about exams, found the volume of homework difficult to deal with, attended off‐campus tutoring for two or more curriculum subjects per week, and were afraid of being punished by teachers and parents (Hesketh et al. 2010; Sun et al. 2013). All these pressures were strongly related to depressive and anxiety symptoms in Chinese adolescents (Fu, Ren, and Liang 2022; Hesketh et al. 2010; Zhou et al. 2021). Similar associations between academic burden and emotional problems have also been reported in other parts of Asia. For example, adolescents in Nepal facing academic stress are 2.4 times more likely to develop depression compared to their peers without such pressures (Pant et al. 2023). A study in India found that academic stress had direct impact on changes in symptoms of generalized anxiety and panic disorder among adolescents (Trevethan et al. 2022).

To understand why adolescents with higher academic burden experience more severe emotional problems, researchers need to examine possible mediating pathways through which academic pressure influences mental health. Based on the classical stress and coping process model (Lazarus and Folkman 1984), Cervantes and Castro developed a more systematic theoretical framework “stress‐mediation‐outcome model” for guiding mental health research (Cervantes and Castro 1985). The essential elements in the model include: potential stressors, stress appraisal, mediators (internal and external), coping response, and outcomes (shorterm and longterm) (Cervantes and Castro 1985). According to their framework and the topic of our study, the objective academic burden such as excessive study time can be considered as a chronic stressor, while subjective academic stress reflects the individual's appraisal of the potential stressor, that is, whether it is perceived as a demanding event. This initial appraisal can influence internal mediators which refer to cognitive and emotional filters people employ to interpret their experience, such as loneliness. A coping response can be behavioral changes of physical activity and sleep as a result of stress appraisal and also related to internal mediators. The final component is the maladaptive outcomes (e.g., depressive and anxiety symptoms) resulting from the cognitive appraisal and coping processes.

Loneliness is defined as an unpleasant feeling resulting from the discrepancy between expected and actual social relationships (Peplau and Perlman 1982). Students with greater perceived stress experience higher sense of loneliness (Liu and Chen 2020). A review about stress and perceived social isolation indicated that stress may play a cocausal or prodromic role in the development of loneliness (Daniel 2019). As one of the strongest predictors of mental distress, loneliness often leads to more severe symptoms of depression and anxiety in adolescents (McIntyre et al. 2018). However, there is little evidence about the mediating effect of loneliness between academic burden and emotional problems.

Physical activity and sleep are often affected by psychological stress. The association between academic stress and physical exercise appears to be negative (Wunsch et al. 2021). A systematic review based on 168 studies reported that psychological stress generally predicted less physical activity, especially in high‐stress periods such as examination phases (Stults‐Kolehmainen and Sinha 2014). Less physical exercise may result in more severe depressive symptoms as exercise was found to effectively reduce depression scores but had no impact on anxiety scores in an overview of systematic reviews (Das et al. 2016). Sleep quality is also a potential factor influencing the association between academic stress and mental health. Sleep quality is influenced by multiple factors such as stress, and students under significant stress are more likely to suffer from sleep deprivation (Ahrberg et al. 2012). Prolonged insufficient or poor‐quality sleep can worsen symptoms of depression and anxiety. For example, a meta‐analytic evaluation of longitudinal studies revealed that nondepressed people with insomnia had a twofold risk of the onset of depression compared to people with no sleep difficulties (Baglioni et al. 2011). Epidemiological studies reported that sleep disturbances, particularly insomnia, affected 50% of people with anxiety, and that insufficient sleep instigated or further exacerbated anxiety symptoms (Chellappa and Aeschbach 2022).

As stated above, loneliness, physical activity and sleep may work together as part of the cognitive appraisal and coping processes in the stress‐outcome relationship. Investigating these mediators simultaneously is necessary because they are essential components in the “stress‐mediation‐outcome model.” In addition, these factors are interrelated and may influence each other in complex ways. Loneliness can lead to reduced motivation for physical activity, as socially isolated individuals often withdraw from social engagement activities, including exercise which may create more opportunities to meet people. Conversely, engaging in regular physical activity may enhance social interactions and reduce feelings of loneliness (Pels and Kleinert 2016). Furthermore, a meta‐analysis reported a medium‐sized correlation between loneliness and sleep disturbance, although the directionality remains unclear (Griffin et al. 2020). Additionally, exercise can improve sleep quality or duration regardless of the mode and intensity of activity (Dolezal et al. 2017; F. Wang and Boros 2021). These complicated relationships suggest that it may be useful to examine the potential mediators simultaneously, which would best fit into the Cervantes and Castro's theoretical framework (Cervantes and Castro 1985).

While research has made strides in understanding the associations between academic factors and adolescent mental health across various cultural and educational settings, their longitudinal associations have not been thoroughly investigated. Many existing studies used cross‐sectional design, and thus further longitudinal research is needed to elucidate the intricate associations. Furthermore, existing research often overlooks potential mediators that could play crucial roles in these associations. To improve mental well‐being in youth, it is critical to investigate mechanisms by which academic factors may relate to adolescent mental health and identify potentially modifiable targets for intervention. The longitudinal cohort study among adolescents in Taizhou, China, offered an opportunity to study the academic factors associated with mental health problems and potential pathways in detail. Our study aimed to estimate the relative contribution of study time, academic stress, and three potential mediators—loneliness, physical activity, and sleep—on depressive and anxiety symptoms. We hypothesized that academic burden would be associated with depressive and anxiety symptoms in adolescents and that loneliness, physical activity, and sleep would mediate the association between academic burden and youth mental health.

## 2 Methods

### 2.1 Study Design and Participants

We conducted a longitudinal cohort study among secondary school students in Taizhou City, Zhejiang Province, China. Employing a multistage cluster sampling method, we selected five districts and counties, including one urban district (Jiaojiang), two county‐level cities (Linhai and Yuhuan), and two counties (Tiantai and Sanmen). Within each district or county, three middle schools and three high schools were randomly selected. Two classes from each grade in each school were chosen to participate in the surveys. The study followed a longitudinal design, with data collection occurring at three time points: April–May 2022 (T1), September–October 2022 (T2), and February–May 2023 (T3). We included students who were in the classes selected, capable of completing questionnaires, and willing to provide online informed consent. All of the participants were invited to complete online surveys via the Wenjuanxing platform (https://www.wjx.cn), which automatically checked the questionnaires for missing values before submission. A total of 2965 participants took part in all the three assessments from T1 to T3, who were included in the current analysis. Table 1 presents the characteristics of participants in the final sample. The mean age of respondents was 15.2 years (SD 1.7), with 1423 (48.0%) being female at baseline (T1). Most of the participants were in public school (68.9%) and had middle or high family economic status (94.8%). The supplementary material details the flow of participants through each wave of the study and compares the characteristics of those who were followed at T2 and T3 with those lost to follow‐up at T2 and T3.

**Table 1:** Characteristics of participants in the analysis sample.

Variables	Overall (*n* = 2965)	Variables	Overall (*n* = 2965)
**T1**		Relationship with father	
Age, years	15.2 (1.7)	Good	2292 (77.3%)
Sex		Normal/poor	673 (22.7%)
Female	1423 (48.0%)	Study time per week (homework and off‐campus tutoring), hours	30.8 (17.9)
Male	1542 (52.0%)	Academic stress	52.6 (11.2)
School		**T2**	
Public school	2044 (68.9%)	Loneliness	
Non‐public school	921 (31.1%)	Relational connectedness	1.5 (0.6)
Parents' marital status		Social connectedness	1.5 (0.6)
Married	2678 (90.3%)	Self‐perceived isolation	1.5 (0.6)
Others	287 (9.7%)	Physical activity	2.0 (2.0)
Family economic status		Frequency of physical activity per week (excluding physical education classes)	2.0 (2.0)
High	422 (19.3%)		
Middle	1654 (75.5%)		
Low	114 (5.2%)	Weekly physical activity, MET‐minutes/week	1,174.2 (1,869.0)
Father's education			
Primary school or lower	357 (12.0%)	Sleep	
Middle school	1181 (39.8%)	Sleep duration	0.5 (0.7)
High school	843 (28.4%)	Sleep disturbances	0.7 (0.6)
College or higher	584 (19.7%)	Sleep latency	0.9 (0.9)
Mother's education		Daytime dysfunction	1.0 (1.0)
Primary school or lower	467 (15.8%)	Sleep efficiency	0.3 (0.8)
Middle school	1137 (38.3%)	Sleep quality	0.8 (0.8)
High school	789 (26.6%)	Sleep medication	0.1 (0.4)
College or higher	572 (19.3%)	**T3**	
Relationship with mother		Depressive symptoms	11.1 (8.2)
Good	2518 (84.9%)	Anxiety symptoms	4.8 (4.3)
Normal/poor	447 (15.1%)		

*Note:* Data are mean (SD) or *n* (%) unless otherwise indicated. MET‐minutes/week stands for metabolic equivalent minutes per week.

The study was approved by the Ethics Committee of Taizhou Central Hospital (2022L‐01‐17), and all methods were carried out in accordance with relevant guidelines and regulations. Informed consent was obtained from all participants and their parents or legal guardians prior to participation in the study.

### 2.2 Measurements

#### 2.2.1 Academic Burden Relevant Variables

(1) Academic stress was measured by the 16‐item Educational Stress Scale for Adolescents (ESSA) (Sun et al. 2011, 2013). The scale contains five domains, including pressure from study, workload, worry about grades, self‐expectation, and despondency. Items were initially created in English and then were translated into Chinese by the development team of the scale. Validated in Chinese students, this scale serves as an appropriate instrument for quantitatively examining academic stress among Asian adolescents. Comprising 16 items (e.g., “I am very dissatisfied with my academic grades”), the scale adopts a 5‐point Likert‐type response format, ranging from 1 (Strongly Agree) to 5 (Strongly Disagree), with a total score range of 16–80. Higher scores indicate greater stress after reverse scoring. The scale demonstrated a Cronbach's α coefficient of 0.90 in our study.
(2) Study time was calculated as the total hours of the following activities per week, including time spent on homework assigned by school teachers, time spent on homework assigned by parents and off‐campus tutors, and time spent on off‐campus tutoring related to school subjects.

#### 2.2.2 Mental Health Outcomes

(1) Depressive symptoms during the last 2 weeks were measured by the 27‐item Children's Depression Inventory (CDI) (Kovacs 1985). The CDI is applicable to individuals aged 7 to 17 years old and has been successfully translated into Chinese and validated for use among children and adolescents in China (Wu et al. 2010; YU and LI 2000). Each of the 27 items can be scored from 0 to 2 (e.g., “I get sad from time to time; I am sad many times; I am sad all the time”), with total scores ranging from 0 to 54. Higher scores indicate more severe depressive symptoms. The scale demonstrated a Cronbach's alpha coefficient of 0.90 in our study.
(2) Anxiety symptoms over the past 2 weeks were assessed by the Generalized Anxiety Disorder‐7 (GAD‐7) (Spitzer et al. 2006). The GAD‐7 is widely utilized for assessing anxiety symptoms, which has been translated into Chinese (Tong et al. 2016) and validated in Chinese adolescents (Sun et al. 2021). It consists of 7 items with a total score range of 0 to 21 (e.g., “Feeling nervous, anxious, or on edge”). Responses are scored 0, 1, 2, and 3 for the categories “not at all,” “several days,” “more than half the days,” and “nearly every day,” respectively. The scale exhibited a Cronbach's alpha coefficient of 0.93.

#### 2.2.3 Mediators

(1) Feelings of loneliness were assessed by the three‐Item UCLA Loneliness Scale (UCLA) (Hughes et al. 2004; Mullen et al. 2019). The UCLA is a validated instrument to assess feelings of loneliness, including items of relational connectedness, social connectedness, and self‐perceived isolation (e.g., “How often do you feel that you lack companionship?”). The items were rated from 1 to 3 (1 = hardly ever, 2 = some of the time, 3 = often), and the total score ranged from 3 to 9, with higher scores indicative of heightened perceived loneliness. The satisfactory reliability and validity of the Chinese Version of the UCLA Loneliness Scale have been confirmed in a variety of Chinese samples including adolescents (Cy et al. 2022; S. Xu et al. 2018). The Cronbach's α of the scale was 0.89, showing good internal consistency in our sample.
(2) Physical activity (PA) was assessed by the frequency of physical exercise per week, and the index of the International Physical Activity Questionnaire–Short Form (IPAQ‐SF) (Craig et al. 2003; Warnimont 2018; Yu, Zhu, and Qiu 2017). The former did not include exercise during physical education (PE) lessons, while the latter did. The IPAQ‐SF provided the sum of days and minutes spent engaging in vigorous PA, moderate PA, and walking. The index for each PA modality using metabolic equivalent minutes per week (MET‐minutes/week) was calculated as: MET‐level × min of activity/day × days/week. The calculation was conducted thrice, yielding separate MET‐minutes/week for each modality (walking = 3.3 METs, moderate PA = 4.0 METs, vigorous PA = 8.0 METs). The Chinese version of IPAQ‐SF has been validated in previous research, and exhibited good internal consistency and test‐retest reliability (Qu and Li 2004). In our sample, the Cronbach's α of the PA assessment was 0.64. The sum of these produced total MET‐min/week for each student in the sample, with higher scores indicative of longer time and more intense exercise.
(3) Sleep was measured by the Pittsburgh Sleep Quality Index (PSQI) (C. Wang, Chen, and Zhuang 2013). The PSQI comprises 19 items and can be divided into seven components, including duration of sleep, sleep disturbance, sleep latency, day dysfunction due to sleepiness, sleep efficiency, overall sleep quality, and use of sleep medication (e.g., “During the past month, what time have you usually gone to bed at night?”). Each component is scored on a scale of 0 to 3, with the cumulative score across components yielding the total PSQI score. Higher scores indicate poorer sleep quality. This tool has been validated for diverse populations (Mollayeva et al. 2016). Using the standard translation‐back translation method, the Chinese version of the PSQI ensured the semantic equivalence between the English and Chinese versions of the PSQI (Tsai et al. 2005). The reliability measures in our sample indicated satisfactory internal consistency (Cronbach's α = 0.75).

#### 2.2.4 Covariates

All covariates were based on data at T1. Sociodemographic characteristics included age (years at T1, continuous), gender (female, male), school (public school, non‐public school), family economic status (high, middle, low), parents' marital status (married, others), parents' education (primary school or lower, middle school, high school, college or higher) and relationship with parents (good, normal or poor). The selection of covariates was based on past literature documenting their associations with adolescent educational burden, mental health problems and mediators.

### 2.3 Statistical Analysis

For each participant, we included data on two academic burden exposures (study time and academic stress, measured at each participant's first wave T1), three latent mediators (loneliness, physical activity and sleep, measured at each participant's second wave T2), and two outcomes (depression and anxiety, measured at each participant's final wave T3). We also included confounders related to the mediators and outcomes (Supporting Information Table S1).

We analyzed the distribution of all variables using mean, SD, frequency, and percentage. There were no missing data for the participants involved in the analyses as the Wenjuanxing platform automatically checked for missing values and reminded the participants before submission. In primary analyses, we modeled each of the mediators as continuous latent factors. Details of the factors used in the latent mediators, selected based on previous research (Craig et al. 2003; Hughes et al. 2004; Mollayeva et al. 2016; Mullen et al. 2019; Wang, Chen, and Zhuang 2013), are provided in Table 2.

**Table 2:** Summary of variables.

Variables	Details of variables
**Academic burden relevant variables (measured at T1)**	
Study time	The total hours of activities(time spent on homework assigned by school teachers, time spent on homework assigned by parents and off‐campus tutors, and time spent on off‐campus tutoring related to school subjects)per week.
Academic stress	Scores of ESSA, where higher scores indicate higher academic stress.
**Mediators (measured at T2)**	
Loneliness	Latent measure of loneliness using three items of UCLA (relational connectedness, social connectedness, and self‐perceived isolation).
Physical activity	Latent measure of frequency of physical exercise per week(excluding exercise during PE lessons), and the index of IPAQ‐SF (MET‐minutes/week)(including exercise during PE lessons).
Sleep	Latent measure of sleep quality using seven components of PSQI (sleep duration, sleep disturbances, sleep latency, daytime dysfunction, sleep efficiency, sleep quality, and sleep medication).
**Mental health outcomes (measured at T3)**	
Depressive symptoms	Scores of CDI, where higher scores indicate more severe depressive symptoms.
Anxiety symptoms	Scores of GAD‐7, where higher scores indicate more severe anxiety symptoms.
**Covariates**	
Age, sex, type of school, family economic status, parents' marital status, parents' education level, relationship with parents	

Abbreviations: CDI, children's depression inventory; ESSA, educational stress scale for adolescents; GAD‐7, generalized anxiety disorder‐7; IPAQ‐SF, international physical activity questionnaire‐short form; MET minutes/week, metabolic equivalent minutes per week; PSQI, Pittsburgh sleep quality index; UCLA UCLA loneliness scale.

To understand the associations between variables before testing the full structural model, we analyzed paths between exposures, mediators, and outcomes in separate regression models, controlling for age, sex, type of school, parents' marital status, family economic status, parents' education level, and relationship with parents. Statistical significance was set a priori at *p* < 0.05 with no adjustment for multiplicity.

All hypothesized mediators were included in the final model simultaneously. Multiple mediation was used in the primary analysis due to its greater convenience, precision, and parsimony compared to multiple single mediation models, and it may also help reduce parameter bias caused by omitted variables (Preacher and Hayes 2008). Results are presented as standardized regression coefficients from the structural equation modeling (SEM). The coefficients were interpreted with regard to direction, magnitude, and statistical significance. The extent of mediation was described as the percentage of the total effect of an exposure mediated by a specific indirect effect. Based on previous research (Chyu and Chen 2022; Tuominen‐Soini and Salmela‐Aro 2014; Bergmann, Muth, and Loerbroks 2019), the academic burden was treated sequentially, with study time preceding academic stress. We specified a correlational, rather than a directional, association between loneliness and physical activity, and between loneliness and sleep due to insufficient evidence regarding their directionality (Griffin et al. 2020; Pels and Kleinert 2016). Physical activity was allowed to influence sleep (Dolezal et al. 2017; Stults‐Kolehmainen and Sinha 2014). The residual covariance between the two outcome variables is presented.

Model fit was assessed using root mean square error of approximation (RMSEA), standardized root mean square residual (sRMR), comparative fit index (CFI), and Tucker‐Lewis index (TLI). RMSEA and sRMR values of 0.06 or less indicated a good fit, although values up to 0.08 were considered acceptable (Hoyle and Panter 1995). For CFI and TLI, values greater than 0.8 reflected an acceptable model fit. The SEM was conducted using the lavaan package ver. 0.6‐16 in R version 4.3.1.

In sensitivity analyses, we separately conducted three mediation models with the dimensions or items of each latent mediator added in a single structural equation model. For example, for the mediating role of loneliness, the three loneliness items were tested as mediators in one model. The models were similar to those in the primary analyses except for the mediating variables to evaluate how individual factors of each mediator affected results.

## 3 Results

**Figure 1:**
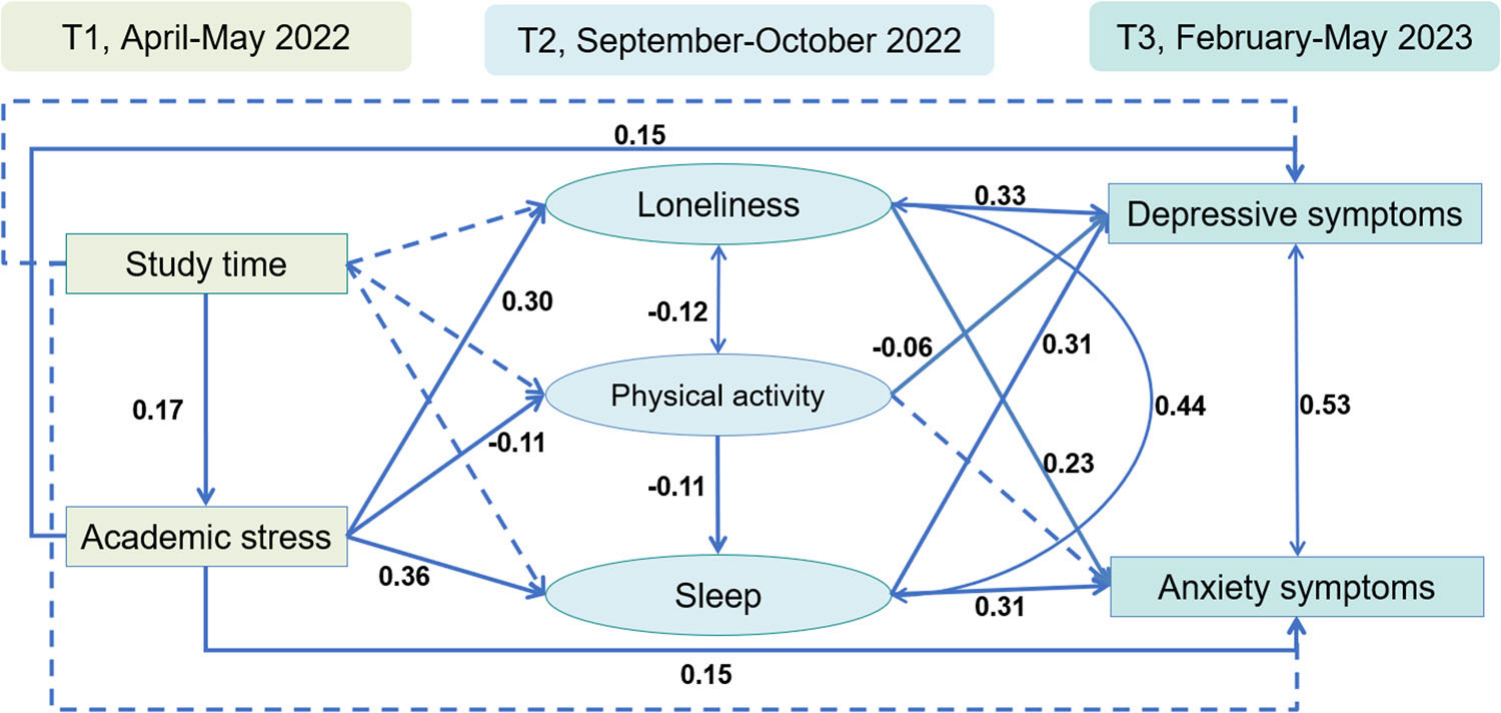
Standardized coefficients for the direct effects for the final full structural model. Note: Single‐headed arrows indicate regression paths, double‐headed arrows indicate covariances, ovals represent latent variables, and rectangles represent measured variables. Coefficients are shown for statistically significant paths, whereas paths with dashed lines were not significant.

Using maximum likelihood estimation with 5000 bootstrapped iterations, the final full structural model exhibited good model fit (RMSEA 0.048, 95% CI 0.046–‐0.050, sRMR 0.035, CFI 0.912, TLI 0.872). In the preliminary analysis (Table 3) and the final model (Figure 1; Table 4), more academic stress was directly associated with higher levels of perceived loneliness, less physical activity, worse sleep, and more severe depressive and anxiety symptoms. In the final model, pathways from academic stress to loneliness and subsequently to depressive symptoms (indirect effect 0.10, 95% CI 0.08–0.11, effect size 26.8%) and anxiety symptoms (indirect effect 0.07, 95% CI 0.05–0.08, effect size 20.6%) were observed. Moreover, physical activity mediated the effect of academic stress on depressive symptoms (indirect effect 0.01, 95% CI 0.002–0.012), accounting for 1.8% of the total effect of academic stress on depressive symptoms. However, physical activity did not significantly mediate the association between academic stress and anxiety symptoms. The hypothesized mediation pathways, proceeding from academic stress to sleep and ultimately to depressive symptoms (indirect effect 0.11, 95% CI 0.09–0.13) and anxiety symptoms (indirect effect 0.11, 95% CI 0.09–0.14), were empirically substantiated, with longitudinal mediation effect sizes of 31.0% and 34.1%, respectively.

**Table 3:** Standardized effects for associations between exposures, mediators, and outcomes.

Associations	Standardized coefficients
Study time > loneliness	0.03 (−0.01, 0.06)
Academic stress > loneliness	0.29 (0.26, 0.33)*
Study time > physical activity	0.02 (−0.03, 0.06)
Academic stress > physical activity	−0.11 (−0.16, −0.07)*
Study time > sleep	0.00 (‐0.03, 0.04)
Academic stress > sleep	0.36 (0.33, 0.40)*
Study time > depressive symptoms	0.04 (0.01, 0.07)*
Academic stress > depressive symptoms	0.36 (0.33, 0.39)*
Loneliness > depressive symptoms	0.55 (0.53, 0.58)*
Physical activity > depressive symptoms	−0.15 (−0.18, 0.11)
Sleep > depressive symptoms	0.55 (0.53, 0.58)*
Study time > anxiety symptoms	0.00 (−0.04, 0.03)
Academic stress > anxiety symptoms	0.33 (0.30, 0.36)*
Loneliness > anxiety symptoms	0.43 (0.40, 0.46)*
Physical activity > anxiety symptoms	−0.08 (−0.11, −0.04)*
Sleep > anxiety symptoms	0.48 (0.45, 0.52)*

* *p* value < 0.05.

> indicates the direction of the regression path, that is, study time predicts loneliness.

**Table 4:** Standardized regression coefficients for the direct and indirect effects for the final full structural model.

	Academic stress	Loneliness	Physical activity	Sleep	Depressive symptoms	Anxiety symptoms
**Mediators**						
Loneliness (direct effect)	—	—	—	—	0.33 (0.30–0.37)*	0.23 (0.19–0.28)*
Physical activity (direct effect)	—	—	—	−0.11 (−0.16 to −0.06)*	−0.06 (−0.10 to ‐0.02)*	0.00 (−0.04 to 0.04)
Indirect effect via sleep	—	—	—	—	−0.03 (−0.05 to −0.02)*	−0.03 (−0.05 to −0.02)*
Sleep (direct effect)	—	—	—	—	0.31 (0.27–0.36)*	0.31 (0.26–0.37)*
**Academic stress**						
Direct effect	—	0.30 (0.26–0.33)*	−0.11 (−0.16 to ‐0.07)*	0.36 (0.32–0.40)*	0.15 (0.11–0.18)*	0.15 (0.11–0.19)*
Total indirect effects	—	—	—	—	0.22 (0.20–0.25)*	0.19 (0.16–0.21)*
Via loneliness	—	—	—	—	0.10 (0.08–0.11)*	0.07 (0.05–0.08)*
Via physical activity	—	—	—	0.01 (0.005–0.02)*	0.01 (0.002–0.012)*	0.00 (−0.00 to 0.00)
Via sleep	—	—	—	—	0.11 (0.09–0.13)*	0.11 (0.09–0.14)*
Via physical activity, and sleep	—	—	—	—	0.004 (0.001–0.01)*	0.004 (0.001–0.01)*
Total effect	—	—	—	—	0.37 (0.34–0.4)*	0.33 (0.30–0.37)*
**Study time**						
Direct effect	0.17 (0.13–0.20)*	0.03 (−0.01 to 0.07)	0.02 (−0.03 to 0.06)	0.00 (−0.04 to 0.04)	0.03 (−0.00 to 0.06)	−0.01 (−0.04 to 0.02)
Total indirect effects	—	—	—	—	0.01 (−0.01 to 0.03)	0.01 (−0.01 to 0.03)
Via loneliness	—	—	—	—	0.01 (−0.00 to 0.02)	0.01 (−0.00 to 0.02)
Via physical activity	—	—	—	0.00 (−0.01 to 0.00)	0.00 (−0.00 to 0.00)	0.00 (−0.00 to 0.00)
Via sleep	—	—	—	—	0.00 (−0.01 to 0.01)	0.00 (−0.01 to 0.01)
Via physical activity, and sleep	—	—	—	—	0.00 (−0.00 to 0.00)	0.00 (−0.00 to 0.00)
Via academic stress	—	0.05 (0.04–0.06)*	‐0.02 (−0.03 to ‐0.01)*	0.06 (0.05–0.08)*	0.02 (0.02–0.03)*	0.02 (0.02–0.03)*
Total effect	—	—	—	—	0.04 (0.00 to 0.08)*	0.00 (−0.04 to 0.03)
**Covariances**						
Loneliness	—	—	−0.12 (−0.17 to −0.07)*	0.44 (0.39–0.48)*	—	—
Depressive symptom	—	—	—	—	—	0.53 (0.49–0.56)*

* *p* value < 0.05.

In the preliminary analysis (Table 3), longer study time was directly associated with more severe depressive symptoms, whereas in the final model, the results showed no significant direct effect of study time on any outcomes or mediators (Figure 1; Table 4). However, longer study time had a positive effect on academic stress (direct effect 0.17, 95% CI 0.13–0.20) and indirect effects via academic stress on loneliness, physical activity, sleep, depressive symptoms, and anxiety symptoms.

Greater loneliness was associated with more severe depressive symptoms (direct effect 0.33, 95% CI 0.30–0.37) and anxiety symptoms (direct effect 0.23, 95% CI 0.19–0.28) (Figure 1; Table 4). Loneliness was correlated with physical activity (coefficient −0.12, 95% CI −0.17 to −0.07) and sleep (coefficient 0.44, 95% CI 0.39–0.48) in the final model. Physical activity had direct effect on depressive symptoms (direct effect −0.06, 95% CI −0.10 to −0.02) but not anxiety symptoms. More physical activity was associated with better sleep (direct effect −0.11, 95% CI −0.16 to −0.06). Sleep had direct effects on depressive symptoms (direct effect 0.31, 95% CI 0.27–0.36) and anxiety symptoms (direct effect 0.31, 95% CI 0.26–0.37).

In our sensitivity analyses, the results from the single mediator models were largely consistent with the final model estimates (Suppoerting Information Table S2). All three items of the loneliness measure (relational connectedness, social connectedness, and self‐perceived isolation) mediated the effects of academic stress on depressive and anxiety symptoms (Supporting Information Figure S2). Frequency of physical exercise excluding PE lessons mediated the associations between academic stress and depressive and anxiety symptoms, but weekly physical activity level including PE lessons (IPAQ‐SF) did not (Supporting Information Figure S3). Academic stress had indirect effects via sleep duration, sleep disturbances, sleep latency, daytime dysfunction, and sleep quality on depressive symptoms, while via sleep duration, sleep disturbances, sleep latency, and daytime dysfunction on anxiety symptoms (Supporting Information Figure S4). The effect sizes for the pathways through daytime dysfunction and sleep disturbances were larger than the other dimensions of sleep (Supporting Information Table S2). The associations between study time and depressive symptoms was not mediated by loneliness, physical activity, and sleep.

## 4 Discussion

Our study conducted a comprehensive investigation into the longitudinal associations between academic burden and symptoms of depression and anxiety, as well as potential mediating pathways among middle and high school students in Taizhou, China. The results indicated that higher academic stress was associated with more severe depressive and anxiety symptoms. Sleep, loneliness and physical activity mediated the association between academic stress and depressive symptoms, accounting for 31.0%, 26.8%, and 1.8% of the total effect of academic stress respectively. Sleep and loneliness also mediated the association between academic stress and anxiety symptoms, with longitudinal mediation effect sizes of 34.1% and 20.6%, respectively. Study time was only associated with the outcomes indirectly via academic stress, reflecting that perceived educational stress may be more relevant to emotional problems than actual study time.

In both preliminary analysis and with structural model analyses, we observed the mediating role of sleep in the associations between academic stress and symptoms of depression and anxiety, underscoring the importance of adequate and high‐quality sleep for psychological well‐being. Multiple factors contributed to the results. On the one hand, academic stress may influence sleep quality through biological mechanisms. Research by Adam et al. suggested that academic stress can disrupt an individual's biological clock, consequently affecting sleep quality (Adam, Snell, and Pendry 2007). According to psychobiological models, academic stress, often marked by worry and rumination, may be related to mental hyperarousal which is a key factor for insomnia (Riemann et al. 2015). The close association between stress and chronic insomnia is also affected by some psychosocial factors. People with insomnia tend to have less satisfying social relationships and more severe loneliness, leading to inadequate coping mechanisms for dealing with stress (Basta et al. 2007; Matthews et al. 2017). On the other hand, the associations between sleep and symptoms of depression and anxiety align with findings from existing literature. A meta‐analytic evaluation of longitudinal studies found that non‐depressed individuals with insomnia had a twofold risk of developing depression compared to those without sleep difficulties (Baglioni et al. 2011). Similarly, insomnia was found to be a major predictor for the onset of anxiety in a meta‐analysis (Hertenstein et al. 2019). Although the psychophysiological mechanisams underlying these associations are still not clear, researchers suggested that the impairment of sleep‐wake regulating neural circuitries may result in changes in emotional reactivity (Riemann et al. 2010). Moreover, in our sensitivity analyses, several domains of sleep, particularly daytime dysfunction and sleep disturbances, were significant mediators of the associations between academic stress and emotional problems. They may be red flags to target for preventing depression and anxiety among adolescents in future studies.

Loneliness mediated the association between academic stress and symptoms of depression and anxiety, accounting for over 20% of the total effect of academic stress. This suggests that people with higher academic stress had more severe emotional problems in part because they had greater loneliness. Several factors may contribute to the observed associations. Educational expectations, concerns about criticism and punishment, and peer pressure are major sources of stress for Chinese adolescents, particularly those with poor academic performance, who often experience weaker social connections and heightened loneliness (Lan et al. 2023). Students experiencing high levels of academic stress may withdraw from social interactions or perceive a lack of support from peers and teachers, contributing to increased feelings of loneliness (Kristensen 2023). Additionally, previous studies indicated a close association between feelings of loneliness and symptoms of depression and anxiety. Individuals experiencing loneliness are more prone to exhibit symptoms of depression such as low mood, loss of interest, and self‐deprecation (Cacioppo and Hawkley 2009; Heinrich and Gullone 2006). Loneliness is also closely associated with symptoms of anxiety, including worries about the future, social anxiety, and doubts about one's own abilities (Hawkley and Cacioppo 2010; Matthews et al. 2016). Our study extends beyond previous research and found that students who perceived deficits in social connections or experienced feelings of isolation were more vulnerable to the negative effects of academic stress on mental health. The results are consistent with a study in healthcare students and early‐career professionals which reported that loneliness mediated the adverse impact of psychological stress on depressive and anxiety symptomatology during COVID‐19 (Bonilla‐Sierra et al. 2021).

Noteworthy, the mediating role of physical activity in the association between academic stress and mental health outcomes is complex. We found a significant association between reduced physical activity and more severe depressive symptoms. Consistent with previous studies, the risk of depression may be reduced by physical activity partly through the release of neurotransmitters such as dopamine and endorphins (Heissel et al. 2023; Pearce et al. 2022). A meta‐analysis found that if the current physical activity recommendations had been achieved by less active individuals, 11.5% of depression cases could have been prevented (Pearce et al. 2022). Although physical activity mediated the association between academic stress and depressive symptoms, the effect size for the pathway was smaller than those for the other two mediators, indicating that sleep and loneliness may play a more important role in the relationship. In further analysis, we found that frequency of physical exercise per week not including PE lessons mediated the association between academic stress and emotional symptoms. However, academic stress did not significantly affect the outcomes via total MET‐min per week which included exercise during PE lessons. Academic stress might reduce leisure physical activity but had less effect on the total intensity of exercise since PE lesson is a compulsory subject in school. Additionally, despite the potential benefits of physical activity, students may experience less pleasure from exercise when it becomes a task linked to their academic performance (Richards et al. 2015). In a systematic review, leasure‐time physical activity was found to be the most effective domain for improving mental health, while occupational or domestic physical activity was related to worse mental health outcomes (Teno, Silva, and Júdice 2024). In terms of anxiety, however, there was a lack of significant association between physical activity and anxiety symptoms in our primary analysis. The result is consistent with a review which reported that vigorous exercise led to diminished depressive symptoms but had no impact on anxiety among adolescents when compared to no intervention (Das et al. 2016). Both fear and avoidance behavior are main characteristics of anxiety symptoms. During exercise with social opportunities such as PE lessons, adolescents are exposed to situations where they have to challenge themselves and interact with other people. These activites may increase their discomfort and trigger physiological reactions (Anker et al. 2024). This speculation can be partly supported by our sensitivity analyses where frequency of physical exercise excluding PE lessons mediated the association between academic stress and anxiety symptoms, but weekly physical activity level including PE lessons did not.

We observed that study time did not have a significant direct association with the mediators or outcomes, but it was associated with these through academic stress. Students who spend longer hours studying often experience higher academic stress. The study time we measured contained time spent on homework assigned by school teachers, parents and off‐campus tutors, as well as time spent on off‐campus tutoring related to school subjects. When the information and tasks placed on working memory exceed its capacity, cognitive overload can occur, which often triggers the stress response (Bodys‐Cupak et al. 2019). Managing multiple tasks simultaneously can create a sense of not having enough time to complete homework or to perform well academically. In addition, the extra learning tasks assigned by parents and off‐campus tutors reduce the time available for relaxation and social activities, and may impair a sense of autonomy regarding their choices, which in turn increase academic stress. The weaker effect of study time on outcomes suggests that academic stress may be a more effective measure than study time for differentiating between adolescents with different levels of academic burden and may be a stronger predictor of mental health problems than measures of objective workload.

Strengths of this study are three waves of longitudinal data, test of multiple competing hypotheses, and use of validated scales. While there is consistent evidence that adolescents with greater academic burden are more likely to experience emotional problems (Fu, Ren, and Liang 2022; Högberg 2021; Högberg, Strandh, and Hagquist 2020; J. Xu et al. 2023), a notable gap exists in empirically investigating factors mediating the associations. Importantly, our evaluation of potential mediators sheds light on the role of sleep, loneliness and physical activity in being the associations, and provides evidence to support the “stress‐mediation‐outcome model” in Chinese adolescents. This research also fills the gap in the literature and provides information for the development of targeted interventions aimed at mitigating the associations between academic burden and mental health of adolescents.

In spite of these advantages, we should acknowledge several limitations. Firstly, our research was conducted in the specific cultural and educational settings of Taizhou, which may not fully represent the experiences of adolescents in other regions or cultural environments. Secondly, although validated measures were used to evaluate academic stress, meditators and mental health outcomes, potential response bias and self‐report bias might still exist. Thirdly, the pandemic might have some impact on the results. However, our study was conducted during a relatively stable period of the pandemic and schools in Taizhou were reopened before the start of the survey at T1, and thus we did not suppose the effect to be profound. Finally, while factors including loneliness, physical activity, and sleep quality have been identified as mediators influencing the association between academic stress and emotional disturbances, the relationships between study time and mental health problems remain incompletely understood. Further research should be designed to measure other potentially modifiable factors on the pathway from actual study burden to emotional and behavioral problems.

Our study has practical implications for guiding educational policies, intervention development and school management practices for adolescents, especially those in a highly competitive educational environment. Given the association between academic stress and depressive and anxiety symptoms, schools should strengthen psychological health education and provide psychological support services to help students effectively manage stress and promote mental well‐being. Our findings also underscore the responsibility of educational policymakers to carry out reforms to reduce students' academic pressure. This may include reassessing curriculum loads and devising more flexible learning schedules to ensure that academic burden is not excessively heavy. More educational burden reduction programs should be designed and implemented, such as the Double Reduction campaign in China aiming at reducing heavy workloads and excessive off‐campus tutoring for students (Beijing: Ministry of Education of the People's Republic of China n.d). Moreover, our study found key mediators which could be important targets for intervention, including sleep, loneliness and physical activity. Parents and school administrators should strengthen collaboration and offer guidance on sleep management to ensure that students obtain sufficient and high‐quality sleep and reduce daytime dysfunction. More social support and opportunities for social activities should also be provided to enhance social connectedness and reduce loneliness. Apart from exercise during PE lessons, parents and teachers can encourage students to engage in leisure‐time exercise, as it may have a better effect on mental health than compulsory physical activity (Teno, Silva, and Júdice 2024). By recognizing and addressing the impact of academic burden on mental health, policymakers, educators and parents should work together to create a suitable and beneficial educational environment which will lay foundation for adolescents' longterm success and happiness.

## 5 Conclusions

In summary, this study found that poorer sleep, higher loneliness and less physical activity experienced by adolescents with greater academic burden was associated with emotional problems. Our results contribute to a deeper understanding of the complex pathways linking academic burden and mental health outcomes among adolescents. The findings underscore the importance of addressing academic burden and provide valuable insights for the development of comprehensive interventions to protect students' mental well‐being. Apart from direct strategies to reduce study time and alleviate academic stress, effective interventions should also focus on mediating factors. Ensuring high‐quality sleep, mitigating loneliness, and encouraging voluntary physical activity during leisure time are all beneficial to adolescent mental health.

## Author Contributions

JW, XC, and CF designed the study and formulated the outline and structure of the article. JW, ZW, and YY conducted data collection and analysis, as well as drafted the article. TW, HL, and WZ interpreted the results and critically reviewed the article. XC and CF reviewed the article. All authors align with the final article.

## Ethics Statement

The study was approved by the Ethics Committee of Taizhou Central Hospital (2022L‐01‐17), and all methods were carried out in accordance with relevant guidelines and regulations.

## Consent

All participants and parents or legal guardians of the children participating in our study provided informed consent prior to study participation. Informed consent procedures were used to collect all study data.

## Conflicts of Interest

The authors declare no conflicts of interest.

## Supporting information

Supporting information.

## Data Availability

The data that support the findings of this study are available from the corresponding author upon reasonable request.
