# Supplementary material for: Academic Burden and Emotional Problems Among Adolescents: A Longitudinal Mediation Analysis
Source: J Adolesc. 2025 Jan 21;97(4):989–1001. doi: 10.1002/jad.12471 (PMC12128909; doi:10.1002/jad.12471)
Supplement: Supplementary file 1 — Supporting information. [file JAD-97-989-s001.docx]

**Article Title:** Academic burden and emotional problems among adolescents: a longitudinal mediation analysis

**Supplementary Material**

**
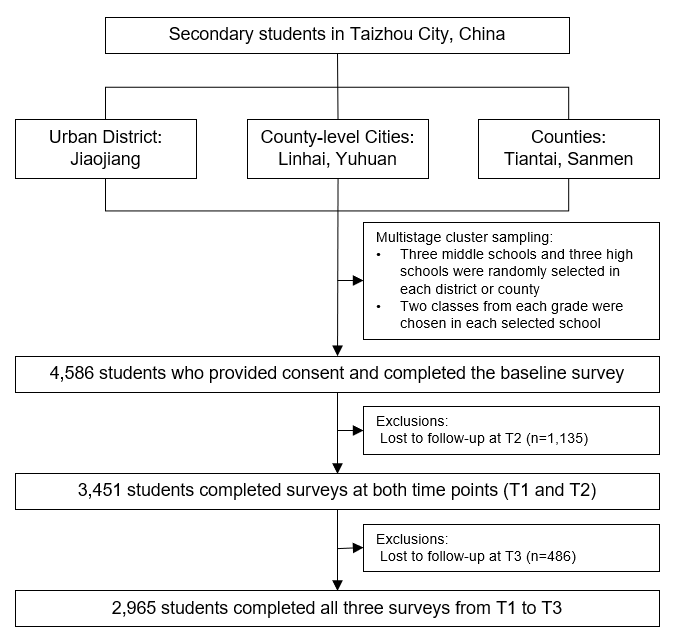
**

# FIGURE S1 Flow chart showing sample size included in the study

# TABLE S1 Participant characteristics between those followed at T2 and T3 and those lost to follow-up at T2 and T3

|  | Lost to follow-up at T2 and T3 | Followed at T2 and T3 |
| --- | --- | --- |
| N | 1621 | 2965 |
| Age, years | 15.8 (1.6) | 15.2 (1.7) |
| Sex |  |  |
| Female | 734 (45.3%) | 1423 (48.0%) |
| Male | 887 (54.7%) | 1542 (52.0%) |
| School |  |  |
| Public school | 1075 (66.3%) | 2044 (68.9%) |
| Non-public school | 546 (33.7%) | 921 (31.1%) |
| Parents' marital status |  |  |
| Married | 1414 (87.2%) | 2678 (90.3%) |
| Others | 207 (12.8%) | 287 ( 9.7%) |
| Family economic status |  |  |
| High | 272 (16.8%) | 422(19.3%) |
| Middle | 1,233 (76.1%) | 1,654(75.5%) |
| Low | 116 ( 7.2%) | 114 (5.2%) |
| Father’s education |  |  |
| Primary school or lower | 239 (14.7%) | 357 (12.0%) |
| Middle school | 723 (44.6%) | 1,181 (39.8%) |
| High school | 412 (25.4%) | 843 (28.4%) |
| College or higher | 247 (15.2%) | 584 (19.7%) |
| Mother’s education |  |  |
| Primary school or lower | 325 (20.0%) | 467 (15.8%) |
| Middle school | 667 (41.1%) | 1,137 (38.3%) |
| High school | 394 (24.3%) | 789 (26.6%) |
| College or higher | 235 (14.5%) | 572 (19.3%) |
| Relationship with mother |  |  |
| Good | 1,332 (82.2%) | 2,518 (84.9%) |
| Normal/poor | 289 (17.8%) | 447 (15.1%) |
| Relationship with father |  |  |
| Good | 1,247 (76.9%) | 2,292 (77.3%) |
| Normal/poor | 374 (23.1%) | 673 (22.7%) |
| Study time per week (homework and off-campus tutoring), hours | 33.1 (21.4) | 30.8 (17.9) |
| Academic stress | 53.9 (11.0) | 52.6 (11.2) |

Data are mean (SD) or n (%) unless otherwise indicated.

# TABLE S2 Effect size and standardized regression coefficients for the indirect effects for academic stress and study time on depressive symptoms and anxiety symptoms via measurement factors of the latent variables (loneliness, physical activity, and sleep)

|  | **Depressive symptoms** | |  | **Anxiety symptoms** | |
| --- | --- | --- | --- | --- | --- |
|  | **Indirect effects** | **Effect size** |  | **Indirect effects** | **Effect size** |
| **Model 1: measurement factors of loneliness** (CFI 0.980, TLI 0.851, RMSEA 0.068, sRMR 0.026) | | | | | |
| **Academic stress** |  |  |  |  |  |
| Via relational connectedness | 0.03 (0.02 to 0.04) ^*^ | 7.9% |  | 0.03 (0.01 to 0.04) ^*^ | 8.0% |
| Via social connectedness | 0.05 (0.03 to 0.06) ^*^ | 12.3% |  | 0.04 (0.02 to 0.05) ^*^ | 11.4% |
| Via self-perceived isolation | 0.05 (0.03 to 0.07) ^*^ | 13.4% |  | 0.03 (0.01 to 0.05) ^*^ | 9.2% |
| **Study time** |  |  |  |  |  |
| Via relational connectedness | 0.00 (-0.00 to 0.01) | - |  | 0.00 (-0.00 to 0.01) | - |
| Via social connectedness | 0.01 (-0.00 to 0.01) | - |  | 0.00 (-0.00 to 0.01) | - |
| Via self-perceived isolation | 0.00 (-0.01 to 0.01) | - |  | 0.00 (-0.00 to 0.01) | - |
|  | | | | | |
| **Model 2: measurement factors of physical activity** (CFI 0.950, TLI 0.695, RMSEA 0.068, sRMR 0.025) | | | | | |
| **Academic stress** |  |  |  |  |  |
| Via frequency of physical exercise per week | 0.01 (0.01 to 0.02) ^*^ | 2.8% |  | 0.002 (0.001 to 0.01) ^*^ | 1.3% |
| Via weekly physical activity level | -0.00 (-0.00 to 0.00) | - |  | 0.00 (-0.00 to 0.00) | - |
| **Study time** |  |  |  |  |  |
| Via frequency of physical exercise per week | -0.00 (-0.00 to 0.00) | - |  | -0.00 (-0.00 to 0.00) | - |
| Via weekly physical activity level | -0.00 (-0.00 to 0.00) | - |  | -0.00 (-0.00 to 0.00) | - |
|  | | | | | |
| **Model 3: measurement factors of sleep** (CFI 0.981, TLI 0.742, RMSEA 0.068, sRMR 0.023) | | | | | |
| **Academic stress** |  |  |  |  |  |
| Via sleep duration | 0.01 (0.01 to 0.02) ^*^ | 3.0% |  | 0.01 (0.00 to 0.01) ^*^ | 2.2% |
| Via sleep disturbances | 0.04 (0.03 to 0.05) ^*^ | 11.4% |  | 0.04 (0.03 to 0.05) ^*^ | 12.2% |
| Via sleep latency | 0.02 (0.01 to 0.03) ^*^ | 4.6% |  | 0.02 (0.01 to 0.03) ^*^ | 5.2% |
| Via daytime dysfunction | 0.05 (0.04 to 0.07) ^*^ | 14.7% |  | 0.05 (0.04 to 0.07) ^*^ | 15.6% |
| Via sleep efficiency | 0.00 (-0.00 to 0.01) | - |  | 0.00 (-0.00 to 0.01) | - |
| Via sleep quality | 0.01 (0.00 to 0.03) ^*^ | 4.0% |  | 0.01 (-0.00 to 0.02) | - |
| Via sleep medication | 0.00 (-0.00 to 0.01) | - |  | 0.00 (-0.00 to 0.00) | - |
| **Study time** |  |  |  |  |  |
| Via sleep duration | 0.00 (-0.00 to 0.01) | - |  | 0.00 (-0.00 to 0.01) | - |
| Via sleep disturbances | 0.00 (-0.00 to 0.01) | - |  | 0.00 (-0.01 to 0.01) | - |
| Via sleep latency | 0.00 (-0.00 to 0.00) | - |  | 0.00 (-0.00 to 0.00) | - |
| Via daytime dysfunction | 0.00 (-0.00 to 0.00) | - |  | 0.00 (-0.01 to 0.00) | - |
| Via sleep efficiency | 0.00 (-0.00 to 0.00) | - |  | 0.00 (-0.00 to 0.00) | - |
| Via sleep quality | 0.00 (-0.00 to 0.00) | - |  | 0.00 (-0.00 to 0.00) | - |
| Via sleep medication | 0.00 (-0.00 to 0.00) | - |  | 0.00 (-0.00 to 0.00) | - |

^*^ p value <0.05.


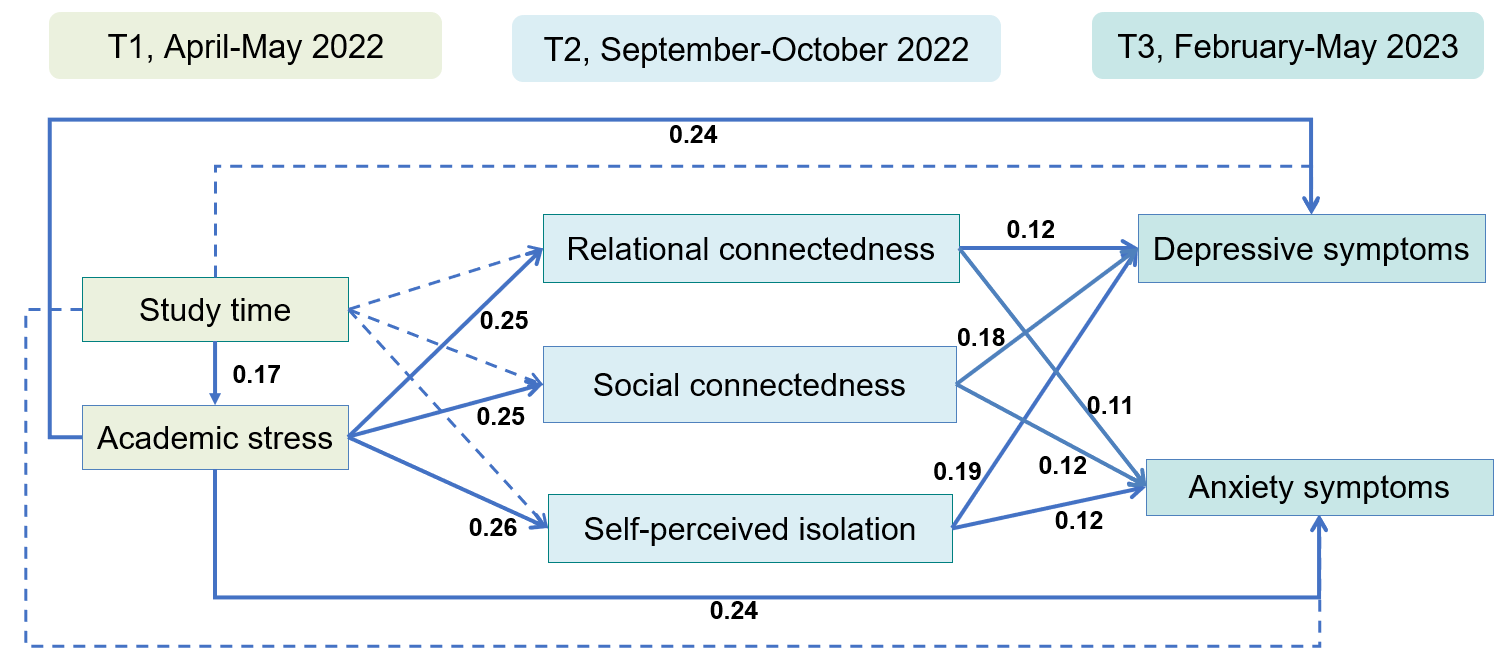


# FIGURE S2 Standardized coefficients for the direct effects for model 1 (loneliness mediators). Note: Single-headed arrows indicate regression paths, and rectangles represent measured variables. Coefficients are shown for statistically significant paths, whereas paths with dashed lines were not significant.


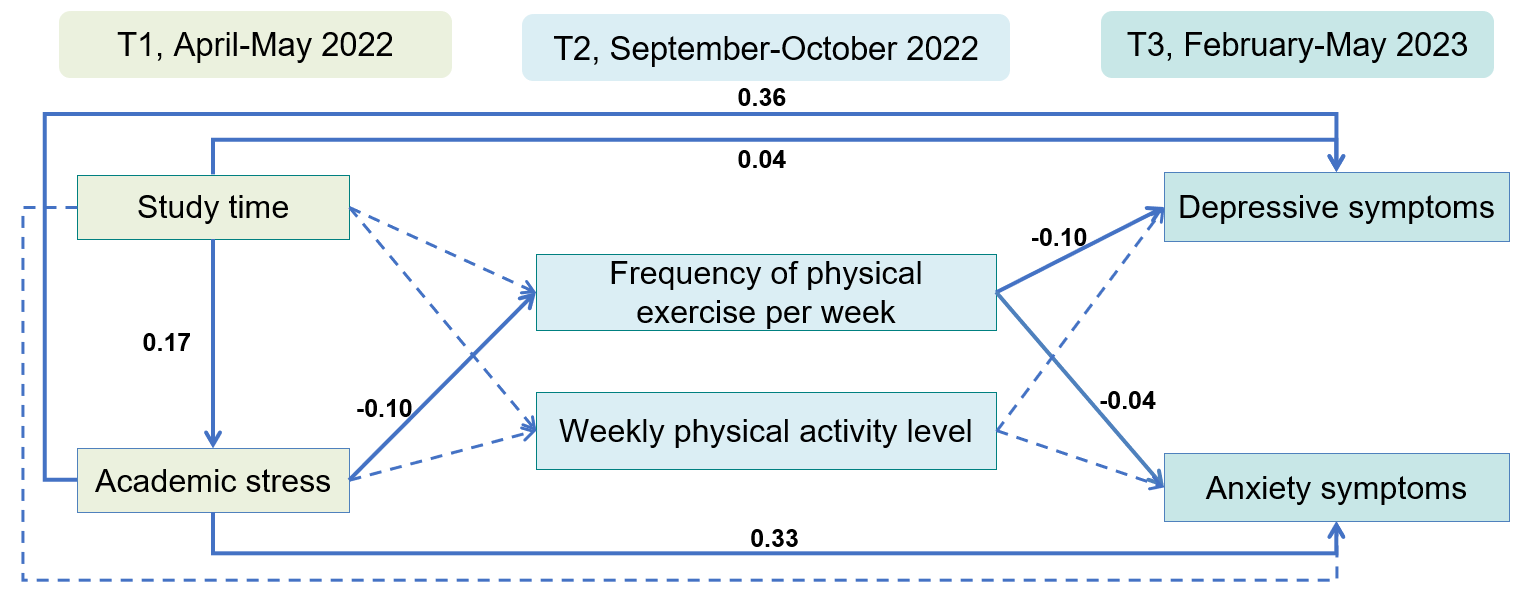


# FIGURE S3 Standardized coefficients for the direct effects for model 2 (physical activity mediators). Note: Single-headed arrows indicate regression paths, and rectangles represent measured variables. Coefficients are shown for statistically significant paths, whereas paths with dashed lines were not significant.


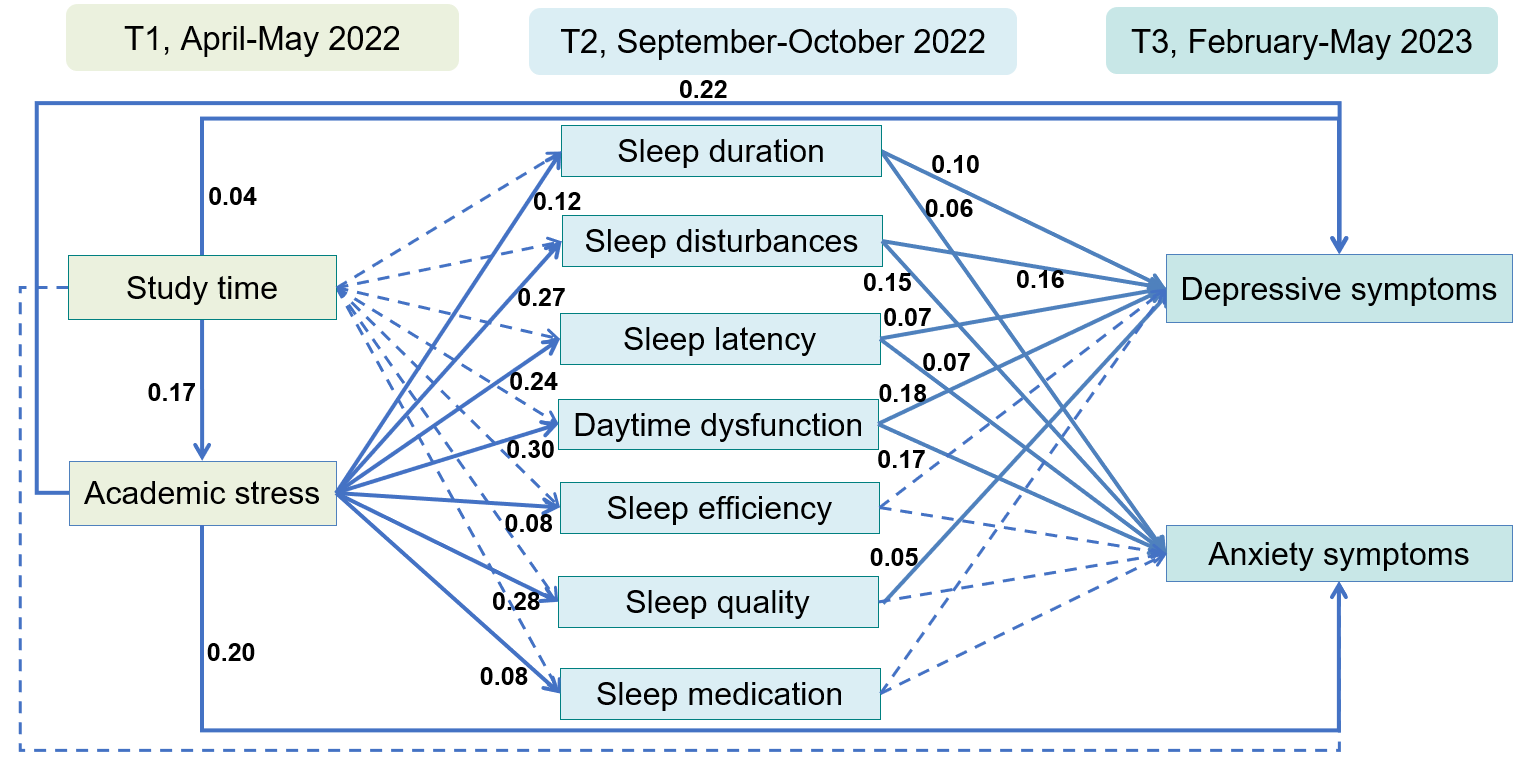


# FIGURE S4 Standardized coefficients for the direct effects for the model 3 (sleep mediators) . Note: Single-headed arrows indicate regression paths, and rectangles represent measured variables. Coefficients are shown for statistically significant paths, whereas paths with dashed lines were not significant.
